# Supplementary material for: From the Perspective of People with Dementia: Using Creative Qualitative Measures to Assess the Values and Opinions on Freedom and Safety among People Living with Dementia
Source: Healthcare (Basel). 2024 Jul 15;12(14):1412. doi: 10.3390/healthcare12141412 (PMC11276790; doi:10.3390/healthcare12141412)
Supplement: Supplementary file 1 [file healthcare-12-01412-s001.zip › healthcare-3049135-supplementary.pdf]

Supplementary Materials

Supplementary File S1: English translation of the observation schedule.

**General information**	
Date	
Time	
Age residents	
Sex residents	
Setting	
**Observations**	
How do the residents feel? What do you observe about their feelings?	
What is the atmosphere like?	
What are residents talking about?	
What needs/wishes can you perceive?	
Are there any relatives present?	Y/N
Describe the interaction with staff	
Which topics are important to the residents at this moment?	
Describe the non-verbal attitude of the residents	
What topics came up during conversation? (circle)	Hobby's/Wishes and Needs/Interests/Qualities/Performance/Other people/Daily matters/Dreams/Social connections/Activities/Other: …
What other things do you perceive?	
